# Observations on Organic Affections of the Heart

**Published:** 1831-05

**Authors:** R. Wade

**Affiliations:** Member of the Royal College of Surgeons, and Lecturer on Morbid Anatomy, &c.


THE LONDON

### Medical and Physical Journal.

No. 387, VOL. LXVI.] MAY 1831. [No. 59, *New Series.*

"For many fortunate discoveries in medicine, and for the detection of numerous errors, the world is indebted to the rapid circulation of Monthly Journals; and there never existed any work to which the Faculty, in Europe and America, were under deeper obligations than to the 'Medical and Physical Journal of London,' now forming a long but invaluable series."—RUSH.

### ORIGINAL PAPERS, AND CASES,

### OBTAINED FROM PUBLIC INSTITUTIONS AND OTHER AUTHENTIC SOURCES.

### ORGANIC AFFECTIONS OF THE HEART.

*Observations on Organic Affections of the Heart.* By R. WADE, Esq., Member of the Royal College of Surgeons, and Lecturer on Morbid Anatomy, &c.

IN the following observations, those of my readers who have more particularly investigated the organic affections of the heart, may perhaps find but little novelty; but as a faithful record of the phenomena observable in such affections, and of the mode of treatment found to tend most to their relief, they may not prove altogether uninteresting; and I trust that, to students, they will, at all events, be useful, and excite their more profound attention to a subject of such vital importance in their profession.

Notwithstanding the admirable work of CORVISART, on Organic Affections of the Heart, for a length of time after its publication, a great degree of scepticism generally prevailed as to the frequency of their occurrence; and it must have been supposed that many of the diseases which he had so accurately described were merely imaginary. Subsequent experience, however, has gradually confirmed most of the views of this enlightened pathologist; and, although, not long ago, with the exception of ossification of the coronary arteries, structural alterations of the heart were seldom recognised by the profession in this country, yet, at this present time, there are but few morbid anatomists to whom most of them are not familiar; and I believe it will be ultimately evident that Corvisart's assertion of their being the most common of all organic diseases, phthisis pulmonalis excepted, is not very far from the truth. It may appear superfluous to observe, that, before we presume to investigate the deviations from the healthy formation of an organ, it behoves us to be acquainted, as far as possible, with its varied, but still healthy, formation in different persons. It is, I think, the neglect of this previous study that gives rise to such discrepant opinions amongst medical men, at post-mortem examinations, concerning the diseases of the heart. It is undoubtedly difficult to define the precise boundaries of healthy and diseased appearances; yet extensive and accurate observations may enable us ultimately to approach, although possibly never to arrive at, perfection in this point.

The conclusions to which my own dissections have led me (which I have no desire to offer with any degree of confidence, but in the hope that they may be useful, by inducing others to investigate the subject,) are, that the heart of a healthy adult may vary in weight from nine to thirteen ounces: at least, when I have found it to be above this size, it has manifested more or less functional derangement during life. The smallest heart, in a person of mature age, which I have met with in my dissections, weighed four ounces four drachms, and the largest twenty-eight ounces. The left ventricle is usually about twice as thick as the right, and the right auricle rather thinner than the left; the four cavities of the heart nearly correspond in size, generally containing from three to four ounces of blood; the auriculo-ventricular openings should admit of the introduction of the ends of two fingers of a moderate size; the valves, in early life, are transparent, but gradually acquire a milky appearance, and, in advanced age, often become very much thickened. The heart should be of a reddish brown colour, and of a tolerably firm consistence; its external and internal surfaces smooth and shining. The extent to which the stroke of the heart is felt in the chest, will vary according to the formation of the latter: it will, for the most part, be felt more distinctly in a small than in a large chest. In a well-formed person, the heart's action should be but slightly perceptible, either in the erect or horizontal position of the body: if it be laboured or jarring, should it intermit or flutter, there will be reason to fear the existence, or anticipate the occurrence, of organic disease. Unless a patient be in a state of tolerable quietude, both mental and corporeal, it will be in vain to expect to arrive at very accurate conclusions concerning the actual degree of mischief in the heart. As the thickness of the parietes of the auricles and ventricles demands attention, the student should omit no opportunity of ascertaining their size, when no particular signs of disordered function have been observed during life.

An anxious expression of countenance commonly attends severe lesions of the heart, and it has been very generally regarded as an important diagnostic sign. Persons subject to these affections are usually hopeless of being cured, and look forward with melancholy foreboding to a fatal termination of their sufferings; they often imagine there is something mysterious in their symptoms, and that they are not very correctly understood by their medical attendants. It has so happened that I have been much oftener requested by patients with structural disease of the heart, to examine their bodies after death, than by others; which circumstance very early excited my attention.

An investigation of the various changes of structure in the heart and pericardium, will soon convince us of their complicated nature; and although, in books, these, with their characteristic symptoms, are separately described, the futility of such distinction will soon be apparent. General descriptions of disease are frequently very injurious to the student; they appear to be often the result of an abstract and analogical reasoning, (than which nothing can be more fallacious,) derived from some few striking cases. It is only by comparing many similar post-mortem appearances with their different symptomatic denotements, and by classing together those symptoms which were of the most common and constant occurrence, and, in contradistinction, those merely occasional, that we can hope to arrive at any satisfactory conclusion. The labours of pathologists, who are content with faithfully and minutely recording the phenomena of disease, are of much more value than those accompanied by speculative opinions, which are only calculated to mislead the many, few of whom have sufficient opportunities of examining diseased structure to be able to detect the errors of others. Intermission of the pulse has been commonly mentioned as a symptom of extensive disease in the valves of the heart: if the reader refers to Case I. in the Appendix, he will observe that the left auriculo-ventricular opening was so small, that it must have considerably obstructed the transit of the blood from auricle to ventricle; yet, during my attendance on this case, the pulse never perceptibly intermitted. I am induced more particularly to mention this circumstance, as, in a discussion occasioned by a paper of mine, read at the Westminster Medical Society, on the Organic Affections of the Heart, much difference of opinion prevailed as to the peculiar alterations of structure which produced the irregularity in its action. Some gentlemen maintained it to be dependent on a diseased state of the aortal, others of the mitral valves; and it was observed that, when the auriculo-ventricular openings were much contracted, the pulse almost invariably intermitted. The facts I witnessed have led me to conclude that the symptoms in question, as well as syncope, instead of being, as they have been so generally regarded to be by those who have written on organic diseases of the heart, amongst their most common accompaniments, are not present in the majority of these cases, if the examination of the circulation be conducted whilst the patient is in a state of quietude. I believe there is scarcely any severe structural lesion of the heart in which the pulse will not be likely to intermit after much bodily or mental exertion. Intermission of pulse, according to my experience, has been more frequently present in the advanced stages of carditis and passive dilatations, than in those cases where the valves were the principal seats of disease.

Syncope, so generally mentioned as a common symptom, has attended but an exceedingly small proportion of the cases of cardiac affection witnessed by myself: it has, however, occasionally been present in ossification of the coronary arteries, in the latter stages of carditis, in passive dilatations, and where the heart was found much attenuated from defective nutrition. Corvisart has this passage: "Dans les cas de rétrécissemens des orifices, d'endurcissement, d'ossification des valvules, ils présentent de la force par momens, quelquefois de la faiblesse, presque toujours de l'intermittence, de l'irrégularité des ondulations, des bruissemens, des frémissemens, dont il est impossible de peindre toutes les variétés." I can only reconcile this passage of Corvisart with my own comparatively very limited observation, by supposing that he must have frequently examined his patients whilst they were under some mental or bodily excitement. As another instance of the fallacy of analogical reasoning, I may mention that MECKEL has particularly described the smallness of the pulse in adherent pericardium, and SENAC the frequent syncope attending it; MORGAGNI observed the pulse to be remarkably small in two cases only. In eight instances of completely adherent pericardium, which I have witnessed, (all in females,) in two the pulse was small, but in these the heart was of a flabby texture; in three, full and jarring, the pericarditis having been combined with hypertrophy of the left ventricle: one example of this disease occurred in a girl only four years of age; in none of them was syncope observed.

In a post-mortem examination at which I was lately present, complete adherence of the pericardium was found to have taken place, although, from the symptoms, no disease of the heart had been suspected. The patient died of cancer uteri, from which she had suffered most severely for two years previously to her death: the whole attention of the medical attendants had been occupied by the uterine affection; indeed, on inquiry, it was afterwards understood that a slight cough, and dyspnœa after exertion, had been the only symptoms which had indicated any affection of the chest. (See Case VIII. in the Appendix.)

From the low degree of sensation with which the heart is endowed, considerable alteration of structure in this organ frequently takes place without the occurrence of any pain to indicate the latent mischief; when, however, the pericardium becomes involved in the disease, more or less pain is usually, but by no means constantly, felt in the region of the heart: in some instances, the patient is scarcely at any time free from it; in others, it is only occasional; in cardiac affections, rheumatic pains are very often experienced in different parts of the body. Corvisart has observed the non-coagulation of the blood after death, occasionally, in organic diseases of the heart: the only instance which has come under my own observation, in which the blood remained quite fluid after life had ceased, is stated in Case I. The same distinguished pathologist asserts, "Après les affections organiques du cœur, le sang est amassé dans les cavités de ce viscère en quantité plus ou moins considerable; je ne sache pas que dans aucun cas de maladie du cœur, il ait jamais été trouvé tout-a-fait vide de sang." The reader will see, in the description of the post-mortem examination, Cases I. III. and VIII., that all the cavities of the heart were quite empty: only one other instance of the kind has fallen under my notice, and in that the blood appeared to be remarkably deficient in every part of the body.

As the prognosis in most organic affections of the heart is extremely unfavorable, it is of great importance to distinguish them from mere functional derangements of that viscus. Palpitations, and other irregular actions of the heart, may be produced by various causes, either moral or physical: if no change of structure be produced, they will generally soon subside, or be much mitigated, provided their causes be removed. Unmarried females, of great nervous excitability, are often subject to these cardiac disturbances: when occasioned, as is frequently the case, by severe mental shocks, they may probably continue for some length of time, without inducing organic lesions. So great is often the nervous susceptibility in these cases, that the entrance of an unexpected friend, or a knock at the door, will quickly raise the pulse thirty or forty beats in a minute; whilst the respiration, between which and the circulation exists so remarkable a sympathy, will be short and hurried. A lady of my acquaintance had functional disturbance of the heart: it is my conviction that no structural disease existed, although an occasional observance of the symptoms would have led to the supposition of more serious mischief. Severe mental suffering was the origin of the disease; the palpitations at first were very strong, but seldom attended by pain; distressing dyspnœa was experienced on the slightest exertion; the pulse, I am informed, was often 140, or upwards, in a minute. The medical attendant of the young lady prescribed digitalis in large doses, half an ounce of the tincture having been taken during the day: the patient was kept under its influence for two or three months, until at length, such was the irritability of the stomach induced by it, the medicine was rejected, even when given in the smallest doses: indeed, medicine of any kind, for a long time afterwards, occasioned a considerable degree of nausea; the slightest agitation would sometimes raise the pulse from twenty to thirty in a minute. Although, after the first few months, the disease considerably diminished in violence, and recurred only at intervals, yet an attack of indigestion, or any mental disquietude, would occasion a return of the palpitations, which were always accompanied by irritability of stomach, which at one time was so considerable, that the greater portion of every thing taken was instantly rejected: it appeared almost useless to attempt to give medicine; for such was her extreme dislike to it, that, besides the certainty of its not being retained in the stomach, the agitation of the patient, if forced to take it, was, of course, of a very injurious tendency. The debility, and frequent occurrence of syncope, at length became alarming; the tongue was clean, pale, and moist: the patient was persuaded to try small doses of quinine, which was instantly rejected; and such was her dread of sickness, that she at last obstinately refused to take any kind of nourishment, and appeared to be unconscious of external impressions, except when spoken to loudly. It was evident to me that the idea of medicine was associated with every thing received into the stomach: I, therefore, assured the patient that, provided she would take nourishment regularly, no medicine should be offered her again during my attendance: although apparently, previous to this promise, unconscious of what was said, she immediately took a teaspoonful of arrowroot, which was frequently repeated, and without any recurrence of vomiting; a striking instance, among many others, of the association of corporeal actions with mental impressions. The palpitations and dyspeptic symptoms returned occasionally for more than two years afterwards, yet, since that time, now nearly five years, the young lady has enjoyed very good health.

Although, in organic affections of the heart, the prognosis is generally unfavorable, yet, occasionally, patients appear to recover from symptoms usually indicating some alteration of structure. It is well known that females, about the age of puberty, are often affected with palpitations, with short and hurried respiration, especially on any exertion: the palpitations are sometimes so strong, and the heart's action so widely felt over the chest, as to lead to the supposition of organic mischief, yet frequently all the symptoms gradually subside. Dr. Parry mentions a singular example of recovery from apparently extensive disease: in this case, the action of the heart was so great as to cause absorption of two ribs; yet, thirty years afterwards, the lady in whom it occurred enjoyed a tolerable state of health. The circulation, at the period when menstruation usually takes place, is often much accelerated, and the heart may become either actively enlarged, from an excess of nutrition, or passively, from the weakness and yielding state of its parietes. As, at this time of life, nutrition is generally active, it is easy to conceive that, as other parts become developed, the heart, if affected by hypertrophy, may gradually lose its preponderance of size; or, when passively enlarged, (which is more frequently the case,) that it may become strengthened by the fibrinous deposition, and thus be enabled to regain its healthy power of resistance to the current of the blood.

When enlargements of the heart take place after the body has attained its full growth, the chance of curing them will, I fear, be slight, although their ill effects may, no doubt, especially in an early stage, be very much controlled by judicious management.

From the pulmonary disturbance which mostly accompanies organic diseases of the heart, especially in enlargements of that organ, they have been often treated as cases of consumption: these mistakes, however, are much less frequent since Corvisart's work on these affections has become generally known. Emaciation to any extent is rather a rare accompaniment of dilatations of the heart in adults, and in many instances large depositions of adeps are observed; which secretion appears to be a means often employed by nature to relieve a distended state of the blood-vessels: as fat contains a large proportion of carbon, may not its formation, also, have the effect of purifying the blood in some degree, by removing its excess of carbon, arising from the imperfect oxygenation of that fluid in the lungs, and its languid circulation in the capillaries? Towards their termination, serum is generally effused in some of the cavities of the body, and anasarca is generally also present, the effusion being often confined to the inferior extremities and the parts of generation: when the scrotum or labia become distended with serum, their occurrence have, in almost every instance that has fallen under my observation, been the heralds of a speedy termination of the sufferings of the patient.

Those affected with enlargements of the heart, particularly when active, are generally the very opposite in conformation to the phthisical, and a very little practical experience is sufficient to enable us to distinguish them.

In confirmation of the remark of Bichat, that osseous depositions, so common to the arterial lining, are never found to affect the common membrane of dark blood, I may observe, that no example of ossification of the valves of the right side of the heart, or pulmonary artery, has occurred to me, although I have found them occasionally thickened.

It is now, I believe, generally admitted that idiopathic hydrothorax is a disease of comparatively rare occurrence, and that most instances of this affection described by authors have been the result of structural disease of the heart, or large blood-vessels: the late Dr. Baillie has fallen into the error of confounding the symptoms of hydrothorax with those of cardiac disease, as I think will be apparent on a reference to his work on Morbid Anatomy. Much difference of opinion prevails as to the cause of many of the post-mortem appearances of disease in the muscular structure of the heart; some authors referring them all to inflammation, others almost denying its existence; amongst whom is Laennec, who, however, allows that the heart itself may be occasionally, although rarely, inflamed in minute spots, but considers the mischief invariably confined to the cellular tissue of the organ. The changes of structure in the heart, which have so often been referred to inflammation, are, a softened and pulpy state of its parietes, which are of a pale yellowish brown, or of a deep red colour, with either indurated or softened texture. It is probable that the pale flabby heart may generally be the result of defective nutrition, although, in one or two instances which I have seen, it was certainly accompanied by inflammation. The deep red-coloured heart may be either the result of a distended state of the cardiac vessels, or of inflammation.

Inflammation of the heart, or pericardium, if unchecked in its early stages by art or nature, so frequently produces irreparable organic mischief, that it demands, when acute, the most active treatment, and, when chronic, unremitting attention. Effective venesection, followed by full doses of opium, combined with calomel, purgatives, antimony, and colchicum, are the best remedies in the acute; the good effects of the latter in inflammation of fibrous structures is well known. Small bleedings, saline aperients with antimonials, the Ung. Antim. Tart. rubbed on the chest, or the occasional application of a blister, will be found useful in the chronic form of the disease: the excitement of a gentle mercurial action will, in some cases, be very beneficial, especially when the mischief appears to be chiefly in the pericardium. We have ample experience of the power of mercury over the capillary or secerning vessels, in relieving their morbid distention, and putting a stop to the inflamed secretions, and probably, at the same time, increasing the action of the absorbents. The mercury should be discontinued, if the patient become debilitated under its use; for, in chronic inflammation, which so often occurs in weak subjects, we should, of course, avoid, as much as possible, diminishing the already too much enfeebled powers of the constitution. The occasional abstraction of small quantities of blood in chronic inflammation of the heart or pericardium, has often the effect of relieving the oppressed vital powers and the distended state of the venous system, without diminishing the force of the heart's action; whilst large bleedings have appeared to me, in most instances, to have eventually done more harm, by weakening the arterial side of the circulation, than good, by taking from the loaded state of the veins; and if it be admitted that the heart and arteries are the most powerful agents in propelling the blood through the veins, and that what is called chronic inflammation consists chiefly in a gorged state of the capillaries, through which their contents pass more slowly than in health, often combined with a debilitated state of the larger branches of vessels, then it will, surely, be also admitted, that we should be exceedingly cautious in employing any remedies, which, however much they may relieve present symptoms, have a tendency to reduce the power of the heart.

When the heart is much diseased, large bleedings will often very quickly be followed by dropsy; the veins become overloaded, the capillary circulation retarded, the vascular system generally in a congested state, and serous effusions are poured into the different cavities of the body; or the patient may become anasarcous; or both these effects may take place at the same time. In most of the organic affections of the heart, bleeding affords such immediate relief, that there is great temptation to have recourse to it: I am well convinced, however, that, generally speaking, it should be adopted with much caution: no temporary advantage should induce us to overlook its ultimate effect upon the disease.

A subacute, or chronic, pericarditis very frequently attacks hysterical girls, and, if it do not ultimately lead to considerable alteration of structure, often proves exceedingly difficult of cure: the pulse is usually accelerated, sometimes full, but more frequently small, and generally hard; an uneasy sensation is almost constantly felt in the pericardiac region; the pain is often severe; the palpitations are occasionally very distressing; the countenance mostly pale and anxious. When the symptoms are urgent, bleeding is the most effectual, and in many cases the only, means of relief.

When the heart acts strongly, I have seen much good result from keeping the patient for a considerable time under the influence of digitalis; and these chronic pericardiac affections, when uncombined with sufficient cardiac mischief to weaken the heart's action, appear to me to be the only ones in which the exhibition of digitalis is desirable; and, should the functions of the stomach become much deranged, its good effects in reducing the force of the circulation will scarcely compensate for the injurious interference with the digestive process.

When mercurial action has been excited in these cases of pericarditis, combined with hysteria, it has generally done harm, by increasing the great nervous susceptibility which is so common an accompaniment of the disease.

Musk in large doses, the different fœtid gums, camphor, hyosciamus, conium, and opium, will frequently be very serviceable, judiciously administered.

In two or three instances, setons afforded more relief than any remedies which were employed. Marriage is often exceedingly beneficial, in cases which had previously a very unpromising appearance.

We must be particular in distinguishing the disease just described from the merely irritable heart, which is often attended with pain, although its periodical character, with other symptoms, will generally render the diagnosis by no means difficult. Inflammation of the heart is, in many instances, accompanied by little or no pain, especially if no dilation or pericarditis be also present. The patient often complains only of uneasiness, or an oppressive sensation at the præcordia. In the latter stage, the extremities are usually cold, the pulse soft and small, and syncope occasionally occurs, especially after much exertion. When these symptoms are present, bloodletting should be adopted with great caution: the abstraction of a few ounces of blood will often materially relieve the labour with which the heart acts, without injuring its power. It is well known that, in the advanced stages of carditis, as in most of the organic affections of the heart, the balance between the arteries and veins is lost, whilst the capillaries are generally in a debilitated state, many parts being gorged with blood; the power of the kidneys seldom being sufficient, for any length of time, to relieve the plethoric state of the system, by removing a sufficient quantity of serum: this fluid is effused into the serous cavities and cellular membrane. In all cases where the serous diathesis prevails, diuretics, diaphoretics, and purgatives, are of great service, and will often very quickly remove the effusion, and consequently afford such complete relief for a time, as to induce those who had not carefully observed cardiac affections to imagine a cure of the disease has been effected. In the last stage, bleeding is seldom admissible, and sometimes proves suddenly fatal, (as in Case v.)

Although, in enlargements of the heart, whether active or passive, much may be done which will materially relieve the patient, I have hitherto had no experience to induce me to believe they can be cured in adults. Active dilatation is generally more under control than passive: the former, if met early by the occasional and free abstraction of blood, by temperance, and by avoiding, as much as possible, excitement of every kind, may often exist for years, without much increasing: the latter, the passive, are usually more rapid in their course towards a fatal termination; for here general bleeding, the most powerful and best remedy in the active, is often, even in a very early stage, of very doubtful utility, from its debilitating the already weakened parietes of the heart; and, supposing the nutritious principle to be active, it is also a question whether we do not render the blood less capable of supplying them with a deposition of fibrin. Should the bleeding be carried to any extent, which is often the case, from the temporary relief obtained by this remedy, it may be expected that the blood will become more serous. When the heart's action is materially weakened, I cannot but think it better to depend chiefly on diuretics and purgatives, which will relieve the circulation by reducing the serous portion of the blood. Should inflammation of the lungs, pleura, or any organ of much importance, be superadded to the cardiac affection, the general abstraction of blood may, of course, be necessary; but, should the pulse be very small and frequent, without hardness, and more especially if intermittent, bleeding must be used (if at all) very sparingly. In these cases, effusion will be found frequently to follow quickly the abstraction of blood; and, in many instances, it will be prudent to rely, for the relief of the inflammation, on the exhibition of calomel and opium, and the application of blisters.

Rheumatic pains are often very troublesome: they are usually best relieved by keeping the surface of the body warm with flannel clothing, by small doses of colchicum and Dover's powder. Digestion is generally much impaired, and, should the flatulency be very distressing, and attended by pain, small doses of hydrocyanic acid, in a little mint-water, will very often afford great relief: if no pain be experienced in the stomach, the tincture of calumbo, or other tonics, may occasionally be given with advantage. It may naturally be expected that the congestive state of the lungs, so common an attendant on organic affections of the heart, will be relieved by the same remedies as are found to be most efficacious in the primary disease. The diet must, of course, be varied according to the powers of the digestive organs, but slops should be avoided, for many and obvious reasons: in most instances, animal food, in small quantities, will be found best.

The various connexions of the par vagum should be well considered, which will throw much light upon many of the numerous symptoms attending affections of the heart. In many organic affections of the heart, the pulse appears to be double: a slight impulse is first communicated to the finger, which is quickly followed by a stronger one, corresponding to the contractions of the auricles and ventricles; and in those cases where this kind of pulse has been observed, (and I have had the opportunity, in these instances, of examination of the body after death,) the mitral valve has been considerably thickened, or the left auriculo-ventricular opening has been contracted; but the double pulse is, I believe, oftener absent than present in those cases where the above-mentioned alteration of structure has taken place.

(To be concluded in our next.)

